# Arterial methemoglobin level predicts therapeutic effectiveness of the PDE5 inhibitors in patients with in idiopathic pulmonary arterial hypertension

**DOI:** 10.1186/1471-2210-11-S1-P71

**Published:** 2011-08-01

**Authors:** Yuichi Tamura, Tomohiko Ono, Takashi Kawakami, Masaharu Kataoka, Motoaki Sano, Toru Satoh, Keiichi Fukuda

**Affiliations:** 1 Keio University School of Medicine, Tokyo, Japan; 2 Kyorin University School of Medicine, Tokyo, Japan

## Background

The PDE5 inhibitors are emerging as novel therapeutic tools in patients with idiopathic pulmonary arterial hypertension (PAH). However, the response to the PDE5 inhibitors varies among patients. The PDE inhibitor augments and prolongs the vasodilator effect of NO. Thus, therapeutic effectiveness of the PDE inhibitors relies on the ability of pulmonary vascular endothelial cells to produce NO. Hemoglobin binds to NO with great affinity and forms methemglobin by oxidation in the erythrocyte. We hypothesized that methemoglobin level is correlated positively with the capacity of NO production in pulmonary vascular endothelial cells and can be used as a biomarker for prediction of therapeutic response to the PDE5 inhibitors.

## Methods

Twenty three idiopathic PAH patients (WHO functional class II or III) who underwent right heart catheterization received the PDE5 inhibitors [sildenafil (n=21), tadalafil (n=2)]. None of them are smokers or taking endothelin receptor antagonist. Arterial methemoglobin level was measured before administration of the PDE5 inhibitors. Hemodynamics were reassessed after a half year observation period (mean following period was 177.5 days).

## Results

Mean methemoglobin level of the patients was 0.7±0.45 % (normal range was 0.2 – 0.6 %). The reduction of mean pulmonary arterial pressures (mPAP) by the PDE5 inhibitors were significantly well correlated with the methemoglobin levels (r=0.46, p=0.027). The patients with high methemoglobin level (≥1.0%) exhibited significantly superior reduction of mPAP than patients with low methemoglobin level (15.5±5.86 mmHg vs 8.18±2.00 mmHg, p=0.04). The reduction of pulmonary vascular resistance also tended to superior in high methemoglobin level group than low level group (6.41±4.08 Wood’s Unit vs 4.56±1.30 Wood’s Unit, p=0.11).

## Conclusion

Measuring methemoglobin level is feasible for prediction of responder and non-responder to the PDE5 inhibitors in patients with idiopathic PAH.

